# A new neutrosophic sign test: An application to COVID-19 data

**DOI:** 10.1371/journal.pone.0255671

**Published:** 2021-08-19

**Authors:** Rehan Ahmad Khan Sherwani, Huma Shakeel, Muhammad Saleem, Wajiha Batool Awan, Muhammad Aslam, Muhammad Farooq

**Affiliations:** 1 College of Statistical and Actuarial Sciences, University of the Punjab, Lahore, Pakistan; 2 Department of Industrial Engineering, Faculty of Engineering—Rabigh, King Abdulaziz University, Jeddah, Saudi Arabia; 3 Department of Statistics, Faculty of Science, King Abdulaziz University, Jeddah, Saudi Arabia; 4 Department of Statistics, Government College University Lahore, Lahore, Pakistan; University of Defence in Belgrade, SERBIA

## Abstract

The Sign test is a famous nonparametric test from classical statistics used to assess the one or two sample averages. The test is practical when the sample size is small, or the distributional assumption under a parametric test does not satisfy. One of the limitations of the Sign test is the exact form of the data, and the existing methodology of the test does not cover the interval-valued data. The interval-valued data often comes from the fuzzy logic where the experiment’s information is not sure and possesses some kind of vagueness, uncertainty or indeterminacy. This research proposed a modified version of the Sign test by considering the indeterminate state and the exact form of the data—the newly proposed sign test methodology is designed for both one-sample and two-sample hypothesis testing problems. The performance of the proposed modified versions of the Sign test is evaluated through two real-life data examples comprised of covid-19 reproduction rate and covid-positive daily occupancy in ICU in Pakistan. The findings of the study suggested that our proposed methodologies are suitable in nonparametric decision-making problems with an interval–valued data. Therefore, applying the new neutrosophic sign test is explicitly recommended in biomedical sciences, engineering, and other statistical fields under an indeterminate environment.

## 1. Introduction

The statistical tools help the researchers highlight the valuable information from the data and precisely hypothesis testing to analyze a data set’s different properties [1]. However, many of such hypothesis tests necessitate that data must follow a normal distribution. Also, a few tests require population variances to be equal. The violation of any of these standard parametric test assumptions leads the researchers to use nonparametric or distribution-free tests [2]. Under Classical Statistics (CS), the Sign test is a nonparametric test applied to test the hypothesis involving matched pairs of a sample or a theory containing nominal data with two categories [3]. Also, this nonparametric test can be applied to test the hypothesis about the population median against a hypothesized value. Many authors worked on the nonparametric sign test; see, for example, refs [4–10].

A theoretical literature review shows that Smarandache [11] introduced a generalization of fuzzy logic named neutrosophic logic. It considers the information regarding the measure of indeterminacy, including the calculation of truthiness and falseness. The neutrosophic statistics developed by Smarandache [12] are useful when the observations of data are in the neutrosophic form. Also, Smarandache and Khalid proved the efficiency of neutrosophic logic; see ref [13]. Chen et al. [24, 25] discussed the advantages of using such methods based on neutrosophic numbers. Several authors in the literature applied the neutrosophic logic as an extension of the classical statistics to deal with the data containing indeterminate parts [14–18]. For a more detailed review of the real-world application of Neutrosophic Statistics, see ref [19, 20]. The fuzzy set system is also a robust and reliable theory to deal with the multiattribute group decision-making problems. The fuzzy set theory is recently expanded by introducing the advanced tool called spherical fuzzy sets (SFSs). Its practical application shows the usage and applicability of such ranking tools used for comparison purposes. In the field of Neutrosophic Statistics, several authors have worked on nonparametric tests designed for the observations containing imprecise or indeterminate part. Aslam [21–23] introduced different statistical tests using Neutrosophic Statistics, such as the tests of homogeneity of variance for uncertain observations, the Kolmogorov-Smirnov test under uncertainty, as well as the goodness of fit test in the presence of imprecise parameters.

The Sign test available in the literature is designed under CS that can only be applied when all the observations in the population or the selected sample are determined/precise. Therefore, the existing sign test cannot be used when the data is measured in the neutrosophic intervals. By exploring the literature and according to the best of our knowledge, there is no such statistical tool present to compare averages of the groups containing data under uncertainty where the assumption of normality does not hold. In this paper, we proposed the sign test under neutrosophic statistics applicable when the exact measurement of the variable is not possible. The objectives of this article are (1) to introduce the neutrosophic sign test; (2) to define the methodology of both one sample and two samples neutrosophic sign test; and (3) to compare the performance of the existing sign test with the proposed test through an application on Covid-19 data set under neutrosophy.

The article is planned as follows: Section 2 briefly describes the methodology to apply the neutrosophic Sign test for a single sample and two samples. In section 3, the proposed neutrosophic sign test has been demonstrated with a descriptive example of the Covid-19 data set for examining its effectiveness and adequacy. It is expected that the suggested nonparametric sign test will efficiently scrutinize the data in the presence of uncertainty and vagueness as compared to the existing sign test under classical statistics. Finally, the results are discussed and generalized with some conclusive remarks.

## 2. Computational method of the sign test under neutrosophic statistics

The main objective of the proposed Sign test is to analyze whether or not the neutrosophic population median has a specified value. The proposed test is applied to the data containing neutrosophic numbers. Furthermore, the test is subcategorized for one sample and two samples. Let *X*_*N*_ = *a*_*N*_+*b*_*N*_*I*_*N*_; *X*_*N*_∈[*X*_*L*_, *X*_*U*_] be a neutrosophic number, where *a*_*N*_ represents the determinate part and *b*_*N*_*I*_*N*_; *I*_*N*_∈[*I*_*L*_, *I*_*U*_] represents the indeterminate part of the neutrosophic number. Note here that if *I*_*L*_ = 0 then *X*_*N*_∈[*X*_*L*_, *X*_*U*_] reduces to a variable under classical statistics. For more detail about neutrosophic statistics, see ref [11]. The neutrosophic variable *X*_*N*_ represents the neutrosophic sample obtained from the population containing imprecise and uncertain and indeterminate observations. More details regarding neutrosophic statistics can be seen in ref [14–18].

### 2.1 Neutrosophic sign test

The existing Sign test under classical statistics cannot be applied when the measure of indeterminacy is required. The neutrosophic sign test is based on the signs of the observed differences of neutrosophic observations, which include an indeterminate part. It is the most straightforward test that can be used as an alternative to the one-sample t-test. This test is based on the signs (pluses and minuses) of the observed neutrosophic differences. The Neutrosophic sign test is used to test the neutrosophic null hypothesis that the probability of a positive sign is equal to the probability of a negative sign, implying that it tests the hypothesis that the neutrosophic population median *NM*_*N*_, has a specified value, say *NM*_0*N*_, because each neutrosophic observation is equally likely to fall above the neutrosophic median as to below it. If there are two neutrosophic samples, it implies that the two neutrosophic populations are identical. The proposed Neutrosophic sign test will be applicable under the following assumptions:

1. The data consists of uncertain, imprecise, and indeterminate values.
2. The two neutrosophic samples must be mutually independent.

#### 2.1.1 One-sample sign test under neutrosophic statistics

Let **X**_**1N**_**, X**_**2N**_,….,**X**_**nN**_ be a neutrosophic sample that is randomly selected from a neutrosophic population. To perform the neutrosophic sign test, replace each neutrosophic observation with a plus sign if the observation is above the specified neutrosophic median represented by **NM**_**0N**_; [**NM**_**0L**_, **NM**_**0U**_] or replace it with a minus sign if it is below **NM**_**0N**_. If any of the observations with an indeterminate part equals **NM**_**0N**_ then that observation is discarded, and consequently, the sample size reduces. The total number of plus and minus signs are denoted by **n**_**N**_. Suppose that **X**_**N**;_ **X**_**N**_**ϵ**[**X**_**L**_**, X**_**U**_] represents the test statistic which is the number of times the less frequent sign occurs. Under the neutrosophic null hypothesis, the neutrosophic test statistic **X**_**N**_ follows a neutrosophic binomial distribution with parameters ½ and **n**_**N**_. To determine the critical region, we computed the neutrosophic binomial probabilities. In the case of a two-tailed test, these probabilities are added; however, for a one-tailed test, the probabilities in the required tail are added to reach the level of significance. The neutrosophic null hypothesis is accepted or rejected in the usual manner.

In the case of one sample, for the proposed test, the neutrosophic null hypothesis that the population median has a specific value *NM*_0*N*_, say *H*_0*N*_∈[*H*_0*L*_, *H*_0*U*_], and an alternative hypothesis, *H*_1*N*_∈[*H*_1*L*_, *H*_1*U*_] are stated below:

$$H_{0N}\in [H_{0L},\;H_{0U}]:\;Neutrosophic\;population\;median\;{NM}_{N}=NM_{0N}$$

$$H_{1N}\in [H_{1L},\;H_{1U}]:\;Neutrosophic\;population\;median\;{NM}_{N}\neq NM_{0N}(or\;{NM}_{N}>NM_{0N}\;or\;{NM}_{N}<NM_{0N})$$

The neutrosophic null hypothesis will be accepted if the computed value of the test statistic falls within the indeterminacy interval of critical values; otherwise, the neutrosophic alternative hypothesis will be accepted. Let *X*_*N*_∈[*X*_*L*_, *X*_*U*_] are the number of times the less frequent sign occurs and is binomially distributed. Subtract the hypothesized value of neutrosophic population median (*NM*_0*N*_) from all observations of the neutrosophic sample, implying that find the differences *X*_*iN*_−*NM*_0*N*_. Mention a plus sign if the difference is positive and a minus sign it is negative. Ignore zero differences. Suppose that the total number of plus and minus signs are denoted by *n*_*N*_. Denoted by *X*_*N*_, the number of times the less frequent sign occurs and computes the extreme probabilities of the binomial variable *X*_*N*_. The critical region depends on the neutrosophic test statistic, neutrosophic alternative hypothesis, and significance level. The decision rule is applied as usual to accept or reject the neutrosophic null hypothesis.

#### 2.1.2 Two sample sign test under neutrosophic statistics

Suppose that *X*_*iN*_ and *Y*_*iN*_ represent the neutrosophic observations from the first and second samples, respectively. The difference between these two (*X*_*iN*_−*Y*_*iN*_) are represented by a plus sign if *X*_*iN*_>*Y*_*iN*_; by a minus sign if *X*_*iN*_<*Y*_*iN*_ and the pair is d if *X*_*iN*_ = *Y*_*iN*_. Let the total number of plus and minus signs are represented by *n*_*N*_ and the test statistic *X*_*N*_; [*X*_*L*_, *X*_*U*_] represents the number of times the less frequent sign occurs. Then the sampling distribution of *X*_*N*_ is neutrosophic binomial with parameters ½ and *n*_*N*_. The rest of the process is the same as in the one-sample neutrosophic sign test. If the sample sizes are not equal, some of the observations with their indeterminate part must be discarded.

The null hypothesis under the proposed sign test for two samples case is described as:

*H*_0*N*_: The two neutrosophic populations are identical, or they have equal medians, *NM*_1*N*_ = *NM*_2*N*_. An appropriate alternative hypothesis is used for the test.

For computational purposes, subtract each observation of the second neutrosophic sample from the corresponding neutrosophic observation of the first sample or find the differences *X*_*iN*_−Y_*iN*_. If the difference is positive, write a plus sign or otherwise write a negative sign if the difference is negative.

### 3. Application of neutrosophic sign test

In this section, we applied the proposed methodology of the neutrosophic sign test on real-life data. The data set contains the 79 observations of the coronavirus reproduction rate in Pakistan from October 2020 to December 2020. For better representation, neutrosophy is introduced in the data and presented in Table 1. The neutrosophic sign test is applied to analyze the assumption that the reproduction rate of coronavirus, specifically in Pakistan, is 1.05.

**Table 1:** Reproduction rate of coronavirus in Pakistan.

[1.02, 1.04]	[1.06, 1.1]	[1.22, 1.26]	[1.3, 1.33]	[1.17, 1.21]	[1.04, 1.08]
[1.03, 1.05]	[1.07, 1.14]	[1.23, 1.25]	[1.29, 1.35]	[1.16, 1.2]	[1.03, 1.06]
[1.03, 1.04]	[1.07, 1.12]	[1.24, 1.25]	[1.29, 1.34]	[1.14, 1.28]	[1.02, 1.04]
[1.03, 1.05]	[1.08, 1.10]	[1.25, 1.25]	[1.28, 1.35]	[1.13, 1.26]	[1.01, 1.02]
[1.04, 1.06]	[1.1, 1.2]	[1.26, 1.27]	[1.27, 1.3]	[1.12, 1.24]	[1.01, 1.02]
[1.04, 1.05]	[1.11, 1.15]	[1.27, 1.29]	[1.26, 1.3]	[1.11, 1.15]	[1.01, 1.03]
[1.04, 1.06]	[1.13, 1.18]	[1.28, 1.3]	[1.26, 1.26]	[1.11, 1.11]	[1.01, 1.02]
[1.04, 1.08]	[1.14, 1.17]	[1.29, 1.33]	[1.25, 1.27]	[1.1, 1.2]	[1.01, 1.04]
[1.04, 1.07]	[1.15, 1.2]	[1.29, 1.29]	[1.24, 1.27]	[1.09, 1.18]	[1.02, 1.04]
[1.04, 1.07]	[1.17, 1.2]	[1.29, 1.3]	[1.23, 1.26]	[1.08, 1.16]	
[1.04, 1.07]	[1.17, 1.19]	[1.3, 1.3]	[1.22, 1.22]	[1.08, 1.16]	
[1.04, 1.08]	[1.18, 1.2]	[1.3, 1.4]	[1.21, 1.25]	[1.06, 1.12]	
[1.05, 1.09]	[1.2, 1.22]	[1.3, 1.3]	[1.2, 1.4]	[1.05, 1.1]	
[1.06, 1.12]	[1.21, 1.24]	[1.3, 1.3]	[1.18, 1.20]	[1.04, 1.08]	

For the data given in Table 1, the number of positive signs is 55, and the number of negative signs is 22 for the determinate part. However, for the indeterminate part, the number of positives is 67, and the number of negatives is 9. As the neutrosophic sample size is [77, 76], which is sufficiently large, the normal approximation to binomial distribution is used.

$$z_{N}=\frac{X_{N}-\frac{n_{N}}{2}}{\sqrt{\frac{n_{N}}{4}}};\;z_{N}\in [z_{L},\;z_{U}]$$ (1)

where *X*_*N*_∈[*X*_*L*_, *X*_*U*_], *n*_*N*_∈[*n*_*L*_, *n*_*U*_]

Here *n*_*N*_ = [77, 79] and *x*_*n*_∈[*x*_*L*_, *x*_*U*_] = [22, 9];

From (1), we have

$$∴P(X_{N}\leq x_{N})=[P(X_{L}\leq 22),\;P(X_{U}\leq 9)]=P(Z_{N}\leq z_{N})$$

p−value≤[0.0001,0.001]

For above mentioned neutrosophic data, the neutrosophic null and alternate hypothesis is: The neutrosophic median of the reproduction rate of coronavirus is 1.05, which implies that $P[+sign]=P[-sign]=\frac{1}{2}$ against the alternate hypothesis that it is not equal to 1.05: *p*≠1/2 (two-tailed test). Assuming significance level 0.05, the maximum calculated neutrosophic p-value must be greater than the significance level to accept the neutrosophic null hypothesis. The neutrosophic statistic from Table 1 is *X*_*N*_∈[22, 9] while the p-value for the data containing indeterminacy is *p*≤[0.0001, 0.001]. The results suggest that both the determined and indeterminate part of the data does not support the neutrosophic median to 1.05.

### 3.1 Example 2

The application of the proposed neutrosophic sign test for two populations is given with the help of Covid-19 data recorded from Pakistan. Concerning the current prevailing situation of Covid-19, the researchers are interested in testing the assumption that there is a significant difference between male and female daily ICU occupancy of Corona-positive patients. The data is recorded in Table 2, and it is evident to perceive that data has neutrosophy. Therefore, the inferential analysis of data using the Sign test under classical statistics may provide misleading results. For such cases, applying the proposed neutrosophic sign test will be relatively practical and informative. The proposed nonparametric test under neutrosophy for variable X is instigated as follows.

**Table 2:** Necessary calculation for the neutrosophic sign test.

Day	Male *M*_*iN*_[*M*_*L*_, *M*_*U*_]	Female *F*_*iN*_[*F*_*L*_, *F*_*U*_]	Sign
1	[443, 450]	[357, 361]	[+, +]
2	[421, 426]	[352, 365]	[+, +]
3	[436, 450]	[445, 455]	[–,–]
4	[376, 385]	410	[–,–]
5	[458, 458]	[458, 464]	[–,–]
6	[408, 420]	[410, 415]	[-, +]
7	[422, 425]	[463, 470]	[–,–]
8	[431, 440]	[580, 584]	[–,–]
9	[459, 462]	[432, 440]	[+, +]
10	369	379	[–,–]
11	360	370	[–,–]
12	[431, 445]	[584, 589]	[–,–]
13	[403, 415]	[410, 416]	[–,–]
14	[436, 445]	[587, 570]	[–,–]
15	376	415	[–,–]
16	370	[419, 422]	[–,–]
17	443	357	[+, +]
18	445	[467, 472]	[–,–]
19	[358, 365]	[415, 418]	[–,–]
20	450	358	[+, +]

For the data given in Table 2, *n*_*N*_ = [19, 20] and *x*_*n*_∈[*x*_*L*_, *x*_*U*_]; [0≤*x*_*L*_≤5,0≤*x*_*U*_≤7]

$$P(X_{N}\leq x_{N})={}^{n_{N}}C_{x_{N}}{\left(p\right)}^{x_{N}}{\left(q\right)}^{n_{N}-x_{N}};$$ (2)

Where *X*_*N*_∈[*X*_*L*_, *X*_*U*_], *n*_*N*_∈[*n*_*L*_, *n*_*U*_]

From (2), we have

$$∴P(X_{N}\leq x_{N})=[P(X_{L}\leq 5),P(X_{U}\leq 7)]=[0.031,0.131]$$

For the data mentioned above, the neutrosophic null and alternate hypotheses are: The male and female covid-19 patients’ daily ICU occupancy is equal, which implies that $P[+sign]=P[-sign]=\frac{1}{2}$ against the alternate hypothesis that daily ICU occupancy for male and female patients is different: *p*≠1/2 (two-tailed test). Assuming the significance level for this test to be 0.05, the calculated value of the neutrosophic test statistic must be greater than the significance level to accept the neutrosophic null hypothesis. The neutrosophic test statistic and p-value for the data given in Table 2 is *X*_*N*_∈[5, 7] and *p*_*N*_∈[0.031, 0.131]. The neutrosophic form of *X*_*N*_ = 5+7*I*_*N*_; *I*_*N*_∈[0, 0.2857]; where 5 presents the value of test statistic under classical statistics and 7*I*_*N*_ is an indeterminate part with measure of intedetermiancy 0.2857. It is evident to note here that the lower value of the indeterminacy interval indicates the determining part. Comparing the value of the neutrosophic p-value with the significance level, we can conclude that the determining part of the data shows a significant difference in daily ICU occupancy based on gender. Still, the indeterminate part of the data shows no difference in daily ICU occupancy of male and female corona-positive patients.

## 4. Discussion

This section compares the performance of the proposed neutrosophic sign test over the sign test under classical statistics. Recent studies have proved that when data contains indeterminacy or uncertainty, the method that provides the indeterminate intervals performs better and provides more adequate results than the method that provides results in the determined form (see ref [24, 25]). To compare the proposed sign test under uncertainty and the sign test under classical statistics, we consider the same data given in Tables 1 and 2. It is evident to note here that if the observations of uncertainty are not recorded, then the data given in Tables 1 and 2 reduces to the determined part under classical statistics. For instance, for sample 1 given in Table 1, the first value 1.02 represents the determinate part of the indeterminacy interval. The second value of this sample represents the indeterminate part of the interval. From these two examples of Covid-19 data, note that the proposed test, for both one sample and two samples, provides the results in the indeterminacy interval rather than the determined values. Using Eq (1), the p-value of the test statistic is *p*≤[0.0001, 0.001]. At a level of significance of 0.05, the probability of accepting the true null hypothesis is 0.95, while the probability of rejecting the true null hypothesis is 0.05 and measure of indeterminacy/uncertainty associated with test is 0.2857. In the case of the neutrosophic sign test for one sample, the example of Covid-19 data shows that both determined and indeterminate parts are significant, implying that the assumption is not true that the reproduction rate of the Covid-19 in Pakistan is not 1.05. However, in two samples, the existing sign test under classical statistics provides misleading results if the data consists of uncertainty or falseness. Note that the indeterminate part is not significant, showing no difference in the daily ICU occupancy by male and female corona-positive patients under uncertainty.

From this comparison, we can conclude that the proposed nonparametric sign test efficiently analyzes the data in the presence of uncertainty and vagueness compared to the existing sign test under classical statistics. In the presence of ambiguity and vagueness, the existing sign is not the right comparison tool as it can generate misleading results, specifically in the case of two samples. The sign test under classical statistics considers only the determinate part, and the existence of the interval-valued data extends the chances of the inferential decision may vary based on the indeterminate part of the neutrosophic number.

## 5. Concluding remarks

In this paper, we proposed the nonparametric sign test under neutrosophic statistics that is the generalization of the sign test under classical statistics. This test can be used to compare the average of paired observations or group(s) consisting of population or selected samples under uncertainty; the suggested test is easy to apply and simple. The proposed neutrosophic sign test is more effective and edifying when data consists of uncertainty and indeterminacy as this new test results in interval-valued form. The neutrosophic sign test results in an uncertain interval, preferable when the data is measured from the complex system. From the comparison, it is concluded that the sign test under classical statistics may generate misleading results in the presence of uncertainty. For this very reason, the application of the neutrosophic sign test is explicitly recommended in biomedical sciences, engineering, and other statistical fields under uncertainty. Furthermore, more properties of this neutrosophic sign test can be derived through simulation for future research. Also, the power of the proposed test can be compared with the Classic Sign test using the Monte Carlo simulation study.
